# Open versus minimally invasive fixation of thoracic and lumbar spine fractures in patients with ankylosing spinal diseases

**DOI:** 10.1007/s00068-021-01756-3

**Published:** 2021-08-06

**Authors:** Felix C. Kohler, P. Schenk, M. Bechstedt-Schimske, B. W. Ullrich, F. Klauke, G. O. Hofmann, T. Mendel

**Affiliations:** 1 Department of Trauma and Reconstructive Surgery, BG Klinikum Bergmannstrost Halle gGmbH, Merseburger Straße 165, 06112 Halle, Germany; 2 Department of Trauma, Hand and Reconstructive Surgery, Jena University Hospital, Friedrich Schiller University Jena, 07747 Jena, Germany; 3 Research Executive Department, BG Klinikum Bergmannstrost Halle, Merseburger Straße 165, 06112 Halle, Germany

**Keywords:** Spine surgery, Ankylosing spinal disease, Ankylosing spondylitis, Bechterew disease, Forrestier’s disease, Diffuse idiopathic skeletal hyperostosis (DISH), Spine fracture, Posterior fixation, Minimally invasive surgery, Open surgery

## Abstract

**Purpose:**

Posterior multilevel fixation of traumatic instability in ankylosing spinal disease (ASD) can be performed by open surgery (OS) or minimally invasive surgery (MIS). We investigated whether both methods differ based on the reduction results and perioperative parameters.

**Methods:**

In this retrospective cohort study, OS and MIS groups were investigated. The bisegmental Cobb angles and dislocation angles were measured using pre- and postoperative CT images, and the initial malalignment and achieved reduction were calculated. Cut-seam time, calculated blood loss, transfusion number, fluoroscopy time, pedicle screw placement accuracy, duration of ICU stay, in-patient stay, and complications (bleeding, postoperative thrombosis and embolism, and postoperative mortality) were recorded.

**Results:**

Seventy-five ASD patients with spine fractures (Ø 75 ± 11 years, male: 52, female: 23) (MIS: 48; OS: 27) were included in this study. The extent of reduction did not differ in the OS and MIS groups (*p* = 0.465; MIS:− 1 ± 3°, OS:−2 ± 6°). The residual postoperative malalignment angle was not significantly different (*p* = 0.283). Seventy-eight of the implanted screws (11%) showed malpositioning. No difference was found between OS and MIS (MIS, 37 [7%]; OS, 41 [16%]; *p* = 0.095). MIS was associated with less blood loss (OS: 1.28 ± 0.78 l, MIS: 0.71 ± 0.57 l, *p* = 0.001), cut-seam time (MIS: 98 ± 44 min, OS: 166 ± 69 min, *p* < 0.001), and hospital stay (MIS: Ø14 ± 16 d, OS: Ø38 ± 49 d, *p* = 0.02) than OS.

**Conclusion:**

OS and MIS show equally limited performance in terms of the fracture reduction achieved. The MIS technique was superior to OS based on the perioperative outcome. Therefore, MIS should be preferred over OS for unstable spinal injuries, excluding C-type fractures, in ASD patients without neurological impairment.

## Introduction

The most frequent manifestations of ankylosing spinal diseases (ASDs) are Bechterew's disease and diffuse idiopathic skeletal hyperostosis (DISH) [1]. Patients with ASD frequently suffer from loss of bone mineral density (BMD) [2]. Literature has shown the prevalence of osteoporosis of up to 60% [3]. During advanced age, the risk of falling increases in these patients. This results in a 4–5 times higher risk of unstable spinal column injuries following low-energy traumas for ASD patients compared with the normal population [4].

**Fig. 1:**
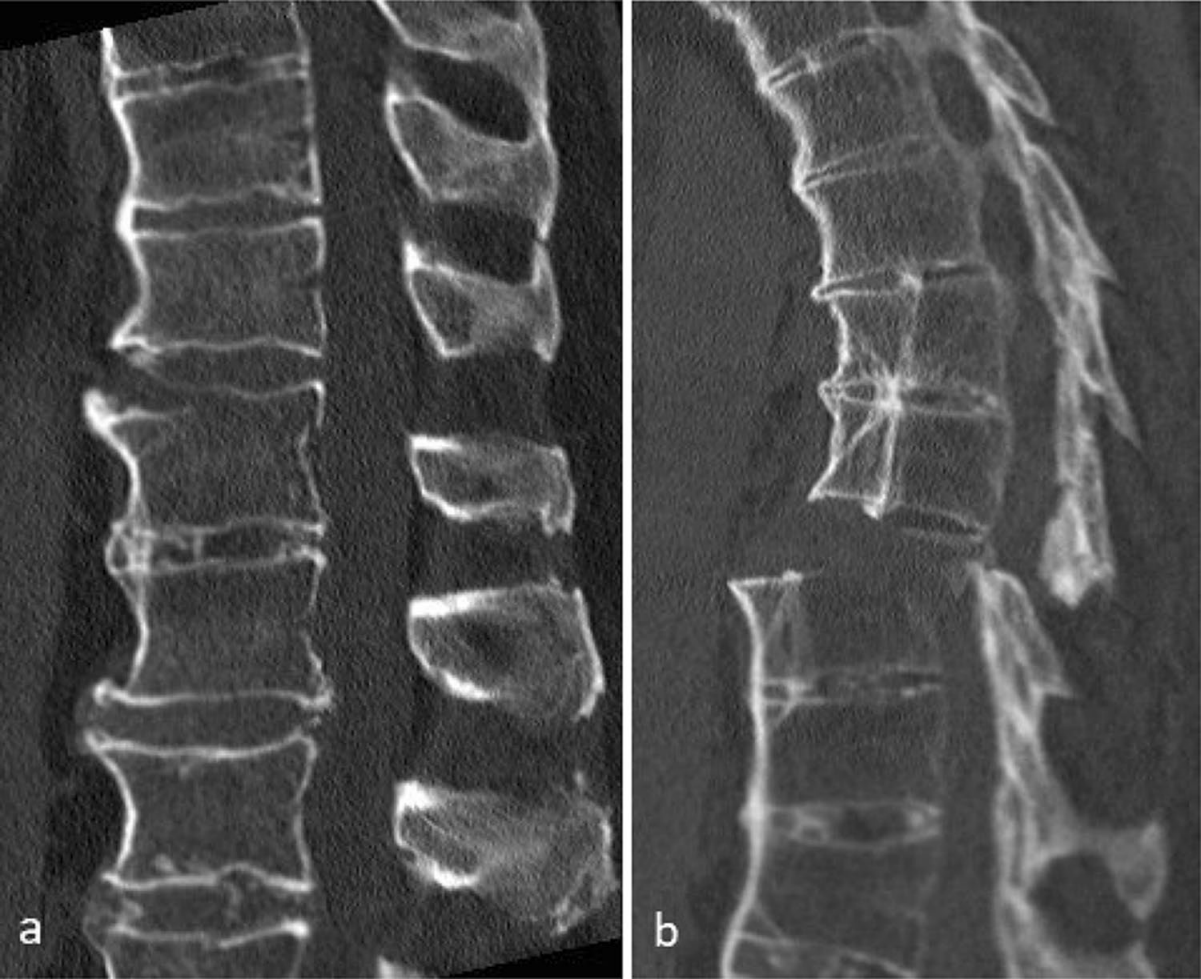
Typical examples of sagittal CT images of a distracted fracture. **a** B3-Type according to AOSpine. **b** C-Type according to AOSpine

Axial ankylosis is characterized by a reduced capacity to distribute the impact energy to adjacent segments, which biomechanically resembles the action of the diaphyseal bone [5]. The long lever effect can, therefore, lead to vertebral fractures in low-energy accidents, such as a fall from a standing or walking position. Owing to the ankylosing ossification of the anterior and posterior elements, most fractures are B- and C-type injuries according to the AOSpine classification system [6]. The most common injuries are of the B3-type B3 injury according to AOSpine, with an incidence of about 74% (Fig. 1) [7]. The second most common types are the B1 and B2 types, with a frequency of about 16% [7–9].

Surgical strategies for spine fractures in patients with ASD are controversial. However, multilevel posterior or combined anterior–posterior fixation should be performed [5, 10] to counteract the considerable leverage effect of the stiffened spine. Anatomical fracture reduction should be attempted [5, 11, 12], regardless of a pre-existing pathological sagittal profile, to prevent neural complications or non-union. Owing to the rigidity of the spine and pre-existing kyphotic deformation, fracture reduction is a considerable challenge and needs to be consistently accessed by a complex positioning of the patient on the operating table and direct reduction maneuvers during surgery. Access can be performed through open surgery (OS) or minimally invasive surgery (MIS).

**Fig. 2:**
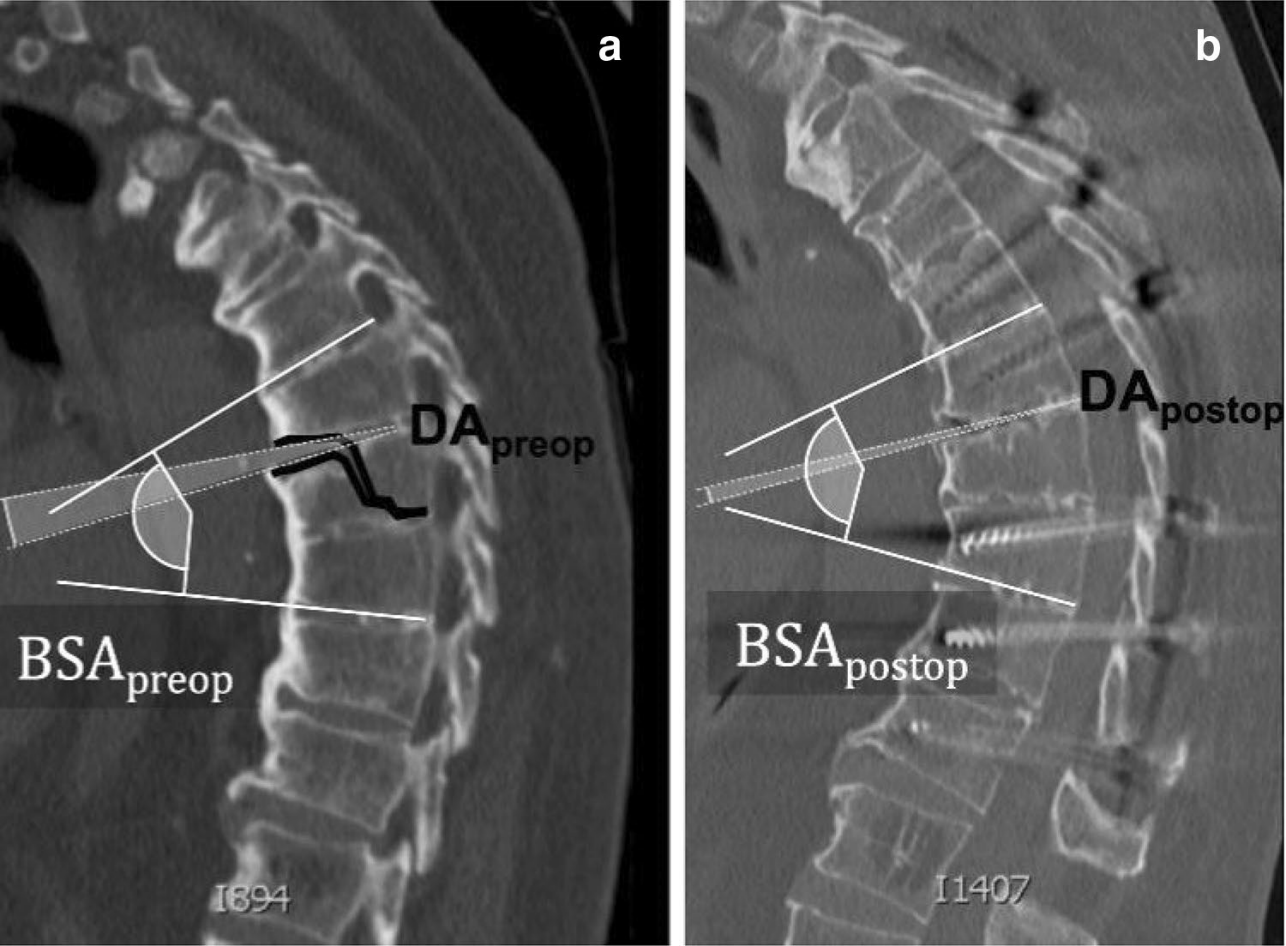
Illustration of the measured parameters of **a** pre- and **b** postoperative sagittal CT scans using the example of a B3-type injury

OS and MIS differ in terms of their technical capabilities. On one hand, depending on the respective system, there exist reduction tools that can be used to correct fracture-related malalignments in all three planes. Compared to MIS, OS might offer technical advantages in terms of reduction due to shorter lever arms for the reduction tools. Furthermore, in OS anatomic landmarks for the pedicle screw, insertion can be located under visual control, whereas with MIS, intraoperative orientation is based on planar fluoroscopy images only. In this respect, OS is expected to have the advantage of lower radiation exposure [13]. These differences contrast with known biological advantages for MIS in perioperative outcome, such as significantly reduce soft tissue damage. Compared with OS, a significantly lower blood loss and lower postoperative complication rate can be expected [14, 15].

In conclusion, MIS and OS differ in terms of their technical capabilities during surgery and in terms of perioperative outcome. From these theoretical considerations, it was hypothesized that the OS technique has advantages over MIS in terms of reduction quality and positioning of pedicle screws, but the MIS technique is superior in perioperative outcome.

To our knowledge, no clinical studies have compared both approaches to multilevel posterior fixation of spine fractures in patients with ASD. This study retrospectively analyzed these aspects in two consecutive ASD cohorts (OS and MIS) treated at a Level I trauma center.

## Materials and methods

The data for the consecutive cohort of ASD patients for 18 years (2002–2019) were retrospectively analyzed; these patients underwent multilevel posterior fixation of fractures of the thoracic and lumbar spine. Different tools for OS and MIS systems were available for the reduction of the fracture-related malalignment. Most often, indirect repositioning manoeuvres such as reduction against pre-bended rods were used to reach adequate alignment, which is possible with both techniques. Alternatively, manufacturer-specific reduction tools were used for both procedures, depending on the instruments used. The hospital information system was searched for patient-related data using an IT-supported search routine based on ICD codes for thoracic and lumbar vertebral fractures and OPS codes for the implantation of a screw-rod system of three or more segments. The diagnosis of ASD was confirmed from the written documentation for the in-patient stay and/or the evidence of typical findings in the CT images. The included patients were assigned to the OS or MIS groups according to the surgical technique applied. Gender, age, BMI, height, weight, BMI, ASA status, and the mechanism of trauma were recorded to describe the population.

The image data were evaluated preoperatively, intraoperatively, and postoperatively by the author (Felix Kohler, MD). For diagnostic purposes, native CT scans were performed. In case of severe injury, total body CT was used. Fracture morphology was assessed using the AOSpine classification system. The initial position of the injured spine was measured as the bisegmental Cobb angle (BSA_preop_) of the fractured segment during preoperative CT sagittal reconstruction. In addition, the extent of the fracture dislocation was quantified. For this purpose, the preoperative dislocation angle (DA_preop_) was measured directly within the fracture gap during the preoperative sagittal CT reconstruction. The estimated individual profile angle (IPA) before trauma was calculated from the difference between the BSA_preop_ and DA. The achieved correction angle (CA) was calculated as the difference between the BSA_preop_ and BSA_postop_. The residual postoperative dislocation angle (DA_postop_) was calculated from the difference between the BSA_postop_ and IPA. Figure 2﻿ provides an overview of the angles described.

Individual bone quality was assessed by calculating the Hounsfield units (HU) on preoperative native spine CT scans [16, 17]. For this purpose, the mean HU values of elliptical regions of interest (ROIs) in three consecutive axial planes of intact adjacent vertebra were calculated [16]. The ROIs were chosen, as large as possible, to exclude the cortical structures of the vertebra. At a mean HU of less than 110 HU, osteoporosis was assumed, as recommended in the literature [16, 18].

The positioning of the pedicle screws was assessed using axial CT scans and classified following the approach of Gertzbein and Robbins [19]. Grade A describes the ideal screw positioning of < 1 mm without perforation of the pedicle wall. A more distinctive pedicular perforation was categorized into the following: B, < 2 mm; C, < 4 mm; D, < 6 mm; E, > 6 mm. Grades A and B were considered to be well positioned. Grades C, D, and E were defined as malpositioned screws. In addition, the need for revision surgery and the presence of inadvertent vascular or neurological injuries were recorded.

The perioperative loss of blood volume (BV_loss_) was calculated based on the calculation methods by Nadler et al. [20] and the perioperative loss of red cells (RC_loss_) was calculated based on the approach by Gombotz et al. [21], taking into account the number of transfused red cell concentrates (RCC).

Surgical complications, such as associated infections, neurological complications, bleeding, postoperative thrombosis and embolism, and postoperative mortality, were recorded.

For process-related assessment of the MIS and OS techniques, both the cut-seam time (CST) and fluoroscopy time were recorded. The need for immediate postoperative intensive care monitoring in the ICU, as well as the total duration of hospital stay, were evaluated in both groups.

The interval-scaled variables were checked for normal distribution using the Shapiro–Wilk test. Differences between MIS and OS were evaluated using unpaired samples. Differences between surgical techniques in fracture morphology, injury level, trauma mechanism, the total amount of implanted pedicle screws, and the amount of malpositioned screws were tested using the Mann–Whitney U test. Crosstable and Pearson’s Chi-squared tests were used to investigate the differences between the groups based on the total number of patients with malpositioned screws. The differences between the two techniques based on reduction quality (DA_postop_ and CA) were verified by separate testing for paired samples for MIS and OS. The impact of bone quality on CA was analyzed using Pearson’s correlation coefficient.

The significance level was set at *α* = 5% (*p* = 0.05). SPSS Statistics V26 (IBM Corp., Armonk, NY, USA) was used for the statistical analyses.

## Results

A consistent dataset of 75 patients who met the inclusion criteria was found. Multi-level posterior fixation was performed using MIS in 48 patients and OS in 27 patients. Neither group showed differences related to the descriptive data (Table2). The distribution of trauma mechanisms (high or low energy) during MIS and OS did not differ. The descriptive data were not significantly influenced by the distribution of the categories based on the AOSpine classification (AOSpine *p* value, Table 2).

**Fig. 3:**
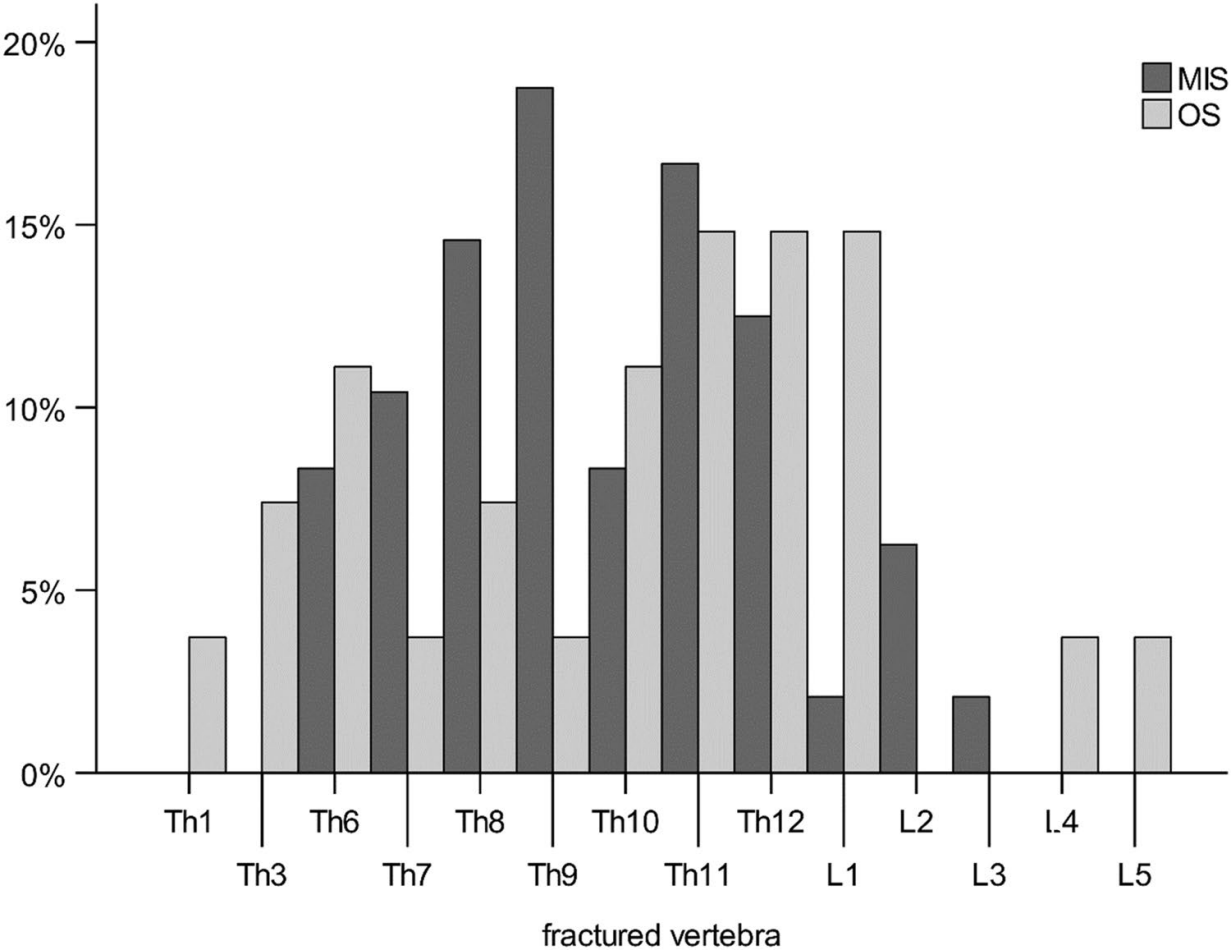
Distribution of the injury levels of the MIS and OS groups

The thoracic spine was affected in 64 patients, whereas the lumbar spine was affected in 11 patients. The distribution of the injured vertebral levels is shown in Fig. 3. No significant differences were found between the MIS and OS groups (*p* = 0.560). In 66 patients, B3-and C-type injuries were detected. Nine patients had B1- and B2-type injuries. B3 was the most common fracture pattern (81%), followed by B1 and B2 (12%) and C (7%). The MIS and OS groups significantly different distributions of injuries according to the AOSpine classification (*p* = 0.028). This may result in differences in dependent variables. Table 2 shows the descriptive data, AOSpine classification, and perioperative process parameters. The table shows (a) the significance of the effect of the AOSpine classification on the parameters (AOSpine *p* value) and (b) the adjusted effect for this variable (*p* value).

**Fig. 4:**
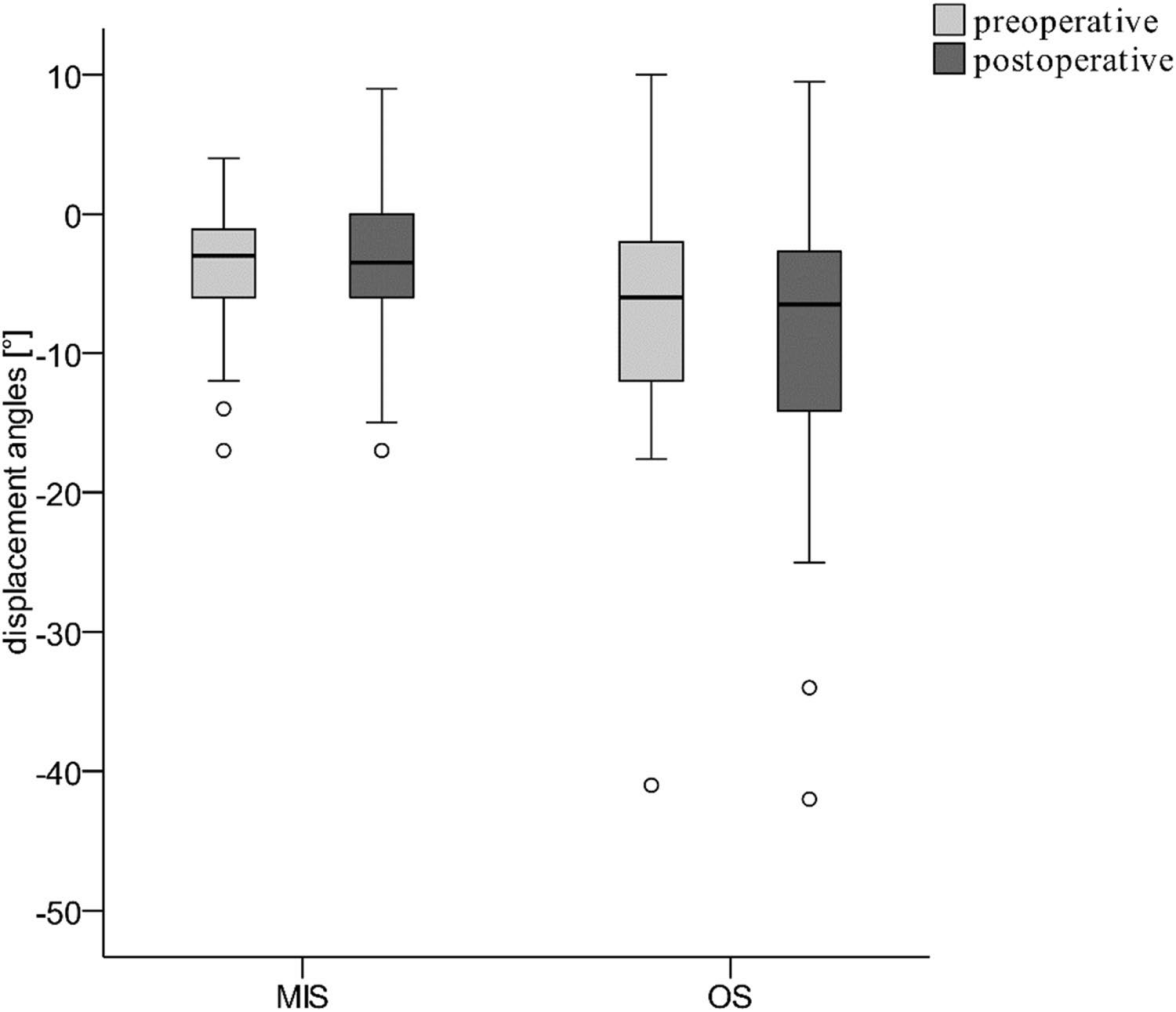
Preoperative (DA) and postoperative residual malalignment angle (RMA) for both groups (MIS and OS)

**Fig. 5:**
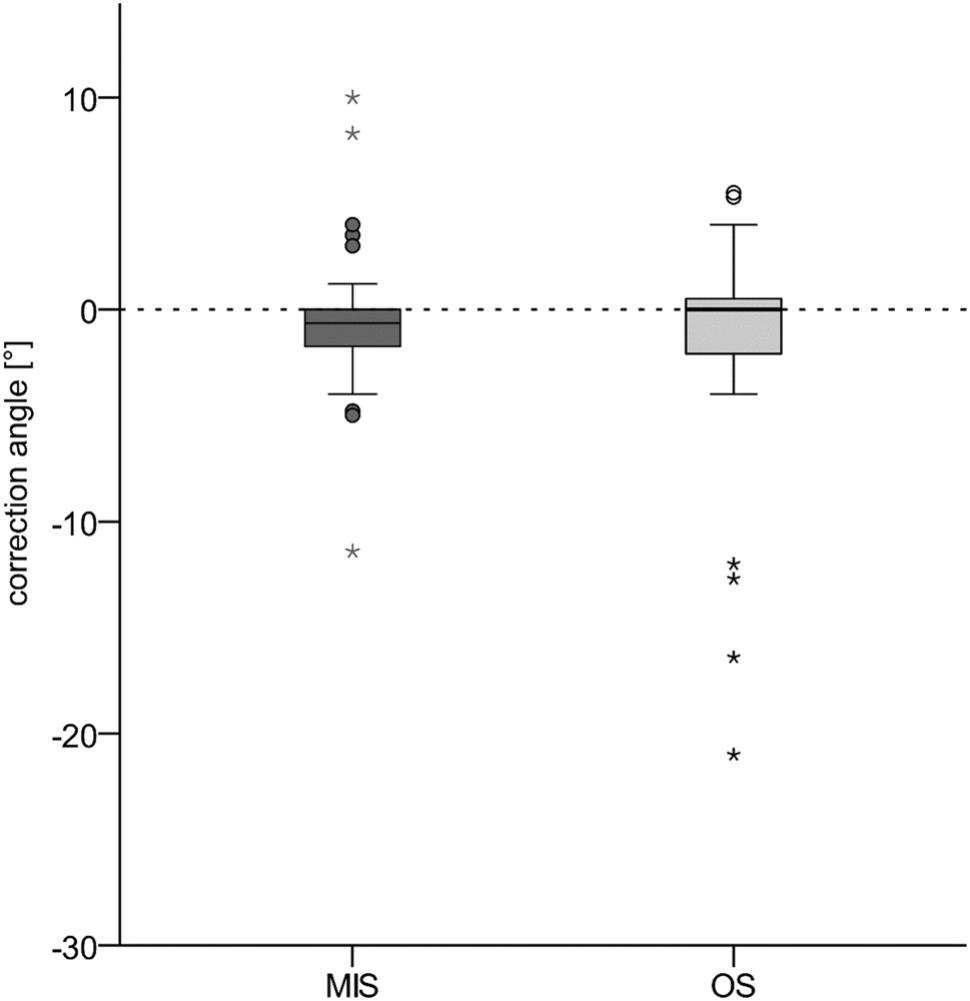
Boxplot comparing the achieved correction angles (CAs) in both groups

The CT imaging did not show a significant difference between the patients in the MIS and OS groups related to the primary fracture-related malposition. The mean DA_preop_ was − 5 ± 7° in the entire cohort (MIS: − 4 ± 4°, OS: − 7 ± 10°, Fig. 4, Table 2). AOSpine had a significant impact (*p* < 0.001). However, the difference was not significant even after adjustment (*p* = 0.059). Postoperatively, the CA did not differ significantly in the MIS and OS groups adjusted according to the AOSpine (MIS, − 1 ± 3°; OS, − 2 ± 6°; *p* = 0.191; Fig. 5, Table 2). The DA_postop_ showed differed across the groups (MIS: − 3 ± 5°, OS: − 10 ± 11°, *p* = 0.001). AOSpine had a significant impact (*p* < 0.001).

The Pearson correlation of HU and CA showed no significance for the separate techniques (MIS, *p* = 0.446; OS, *p* = 0.280) or the entire cohort (*p* = 0.646).

The assessment of the individual bone quality revealed that approximately two-thirds of patients (*p* = 51, 68%) showed HU values below 110 and osteoporosis. There was no difference between the mean bone qualities of the two groups (MIS, 95 ± 55 HU; OS, 91 ± 59 HU; *p* = 0.602). The distributions of the measured HU values within the two groups are shown in Fig. 5.

A total of 736 pedicle screws were used. The individually selected number of screws did not differ across the two surgical techniques. (*p* = 0.083). The distribution of the screw numbers used for fixation constructs is presented in Table 1a. Seventy-eight of the implanted screws (11%) showed critical position grades C, D, or E. No significant difference in frequency between the two groups was found (MIS: 37 [8%], OS: 41 [16%], *p* = 0.095, Table 1 b)). None of the borderline screw positions was associated with neurological effects, but three revisions were performed due to the direct proximity of the screw tip to the aorta (MIS: 1, OS: 2, *p* = 0.293).

**Table 1:** Distribution of the applied internal fixator constructs in relation to the individual number of screws and incorrect positioning in proportion to the total number of screws (a), as well as the number of patients affected and the number of patients in need of surgical revision (b)

Surgical technique, N screws/malpositioned (%)	Internal fixator construct screw no. (superior/inferior to fracture level)						
	4 (2/2)	6 (2/4)	8 (4/4)	10 (4/6)	12 (6/6)	16 (8/8)	Total
(a)							
MIS (48 patients)	–	–	168/11 (7%)	30/3 (10%)	288/23 (8%)	–	486/37 (8%)
OS (27 patients)	4/1 (25%)	12/1 (8%)	112/20 (18%)	10/2 (20%)	96/17 (18%)	16/0 (0%)	250/41 (16%)
Total	4/1 (25%)	12/1 (8%)	280/31 (11%)	40/5 (13%)	384/40 (10%)	16/0 (0%)	736/78 (11%)

Surgical technique	Pedicle screws		Patients with malpositioned screw	Patients needing revision
	Total	Mal-positioned		
(b)				
MIS (48 patients)	486	37 (8%)	20 (42%)	1 (2%)
OS (27 patients)	250	41 (16%)	16 (59%)	2 (7%)
Total (75 patients)	736	78 (11%)	36 (48%)	3 (4%)

The calculated perioperative blood loss was significantly higher in the OS group (1.28 ± 0.78 L) than in the MIS group (0.71 ± 0.57 L, *p* = 0.001, Table 2). Accordingly, within the perioperative circulatory management, the need for transfusions was higher in the OS patients (*n* = 8, 30%) than in the MIS patients (*n* = 5, 10%), and the significance level was just missed (*p* = 0.055). If a transfusion was necessary, patients in the OS group received 2.8 ± 1.4 RCC, and patients in the MIS group received 1.6 ± 0.5 RCC (*p* = 0.072). A significant effect was observed after perioperative administration of crystalline fluids (MIS: 2.0 ± 0.9 L, OS: 3.6 ± 1.4 L, *p* < 0.001, Table 2).

**Table 2:** Descriptive data, AOSpine classification, and process data with p values for both surgical techniques (MIS: minimally invasive surgery, OS: open surgery) and p values adjusted for the AOSpine classification

	AOSpine *P* value	Total	MIS	OS	*P* value
Sex, *n* (%)					1.000
Male		52 (69)	33 (69)	19 (70)	
Female		23 (31)	15 (31)	8 (30)	
Age, (mean ± sd)	0.222	75 ± 11	77 ± 10	72 ± 12	0.077
Body size, (mean ± sd)	0.052	1.72 ± 0.10	1.71 ± 0.10	1.72 ± 0.11	0.901
Body weight, (mean ± sd)	0.093	90 ± 20	88 ± 17	94 ± 25	0.310
BMI, (mean ± sd)	0.445	31 ± 6	30 ± 6	31 ± 6	0.494
HU, (mean ± sd)	0.717	95 ± 55	98 ± 53	91 ± 59	0.598
Affected vertebrae, (mean ± sd)	0.583	10 ± 3	10 ± 2	10 ± 4	0.911
ASA, *n* (%)					0.997
II			13 (27)	7 (26)	
III			26 (54)	15 (56)	
IV			9 (19)	5 (19)	
High-/Low-energy, *n* (%)					1.000
Low energy		57 (81)	38 (81)	19 (83)	
High energy		13 (19)	9 (19)	4 (17)	
AOSpine, *n* (%)					0.028
B1 or B2		9 (12)	4 (8)	5 (19)	
B3		61 (81)	43 (90)	18 (67)	
C		5 (7)	1 (2)	4 (15)	
CST [min], (mean ± sd)	0.242	123 ± 63	89 ± 44	166 ± 69	< 0.001
Fluoroscopy time [s], (mean ± sd)	0.287	2.8 ± 1.8	2.8 ± 1.6	2.9 ± 2.2	0.899
RCC, *n* (%)					0,055
None		62 (83)	43 (90)	19 (70)	
Yes		13 (17)	5 (10)	8 (30)	
RCC, *n* (%)	0.141				0.007
0		62 (83)	43 (90)	19 (70)	
1		2 (3)	2 (4)	–	
2		9 (12)	3 (6)	6 (22)	
5		2 (3)	–	2 (7)	
RCC, (mean + -sd)	0.311	2.31 ± 1.25	1.6 + − 0.5	2.8 ± 1.4	0,072
Crystalline fluids [l], (mean ± sd)	0.592	2.60 ± 1.32	2.05 ± 0.90	3.57 ± 1.42	< 0.001
Blood loss [ml], (mean ± sd)	0.247	916 ± 705	709 ± 572	1.284 ± 778	0.001
DApreop, (mean ± sd)	< 0.001	− 5 ± 7	− 4 ± 4	− 7 ± 10	0.059
DApostop, (mean ± sd)	0.043	− 6 ± 8	− 3 ± 5	− 10 ± 11	0.001
CA, (mean ± sd)	0.326	− 1 ± 5	− 1 ± 3	− 2 ± 6	0.191
Number of screws, (mean ± sd)	0.355	10 ± 2	10 ± 2	9 ± 3	0.103
ICU [d], (mean ± sd)	0.216	5 ± 15	6 ± 16	5 ± 12	0.755
Hospital stay [d], (mean ± sd)	0.027	23 ± 34	14 ± 16	38 ± 49	0.003

*BMI* Body Mass Index, *ASA* American Society of Anesthesiologists, *CST* cut suture time, *RCC* Red Cell Concentrate, *DA* dislocation angle, *CA* correction angle, *ICU* intensive care unit

The CST was significantly shorter in the MIS group (89 ± 44 min) than in the OS group (166 ± 96 min) (*p* < 0.001). The fluoroscopy time did not differ in the groups (MIS: 2.8 ± 1.6 min, OS: 2.9 ± 2.2 min, p = 0.899, Table 2).

Two patients who underwent OS had deep surgical site infections (7%). In contrast, no infections were found in the MIS group (*p* = 0.126). Six patients showed preoperative neurological deficits significantly different from those in the OS group (MIS: 1, OS: 5, *p* = 0.021). No neurological deterioration was observed after surgery. Overall, one patient who underwent OS had bleeding complications and needed surgical revision. Thromboembolic complications did not occur. Inpatient mortality was zero in both groups.

The CST was significantly shorter in the MIS group (89 ± 44 min) than in the OS group (166 ± 96 min) (*p* < 0.001). The fluoroscopy time did not differ in the groups (MIS: 2.8 ± 1.6 min, OS: 2.9 ± 2.2 min, *p* = 0.899, Table 2).

Postoperative intensive medical monitoring in an ICU and/or IMC unit was initiated in 10 (37%) and 23 (48%) patients who underwent OS and MIS (*p* = 0.755). The duration of stay did not significantly differ in the two groups (MIS: Ø12 ± 22 d, OS: Ø13 ± 18 d, *p* = 0.464). However, the entire duration of hospital stay was 2–3 times longer for patients in the OS group than for those in the MIS group (MIS: Ø14 ± 16 days, OS: Ø38 ± 49 days, *p* = 0.003, Table 2).

All the process parameters were not significantly influenced by the distribution based on the AOSpine classification (AOSpine *p* value, Table 2).

## Discussion

This study investigated both the radiological outcome in terms of surgically achieved reduction, recording of bone quality by measuring HU and accuracy of screw positioning and the perioperative outcome in terms of blood loss, complications, process parameters and hospital stay after multilevel posterior fixation of spinal injuries in patients with ASD using OS or MIS technique. The most important findings were that neither technique can improve the trauma related malalignment. In the biological and clinical results, the MIS technique shows clear advantages.

Considering the available literature on our topic, the study cohort of 75 was larger than those of others [5, 7, 22, 23]. The mean age (75 years) and sex ratio (approximately 70:30, m:w) was comparable to the reports in the literature, and was not significantly different in the MIS and OS groups [1, 5, 7]. No comparative data on weight, body size, and BMI distribution were found in the literature after extensive research. These data are difficult to compare in different population groups and they reflect a European collective. Most of the trauma mechanisms involved falling from walking or standing (81%). This confirms the data reported by Westerveld et al.: 66% [7]. The distribution of injury severity in our cohort was consistent with a known pattern. The most common injury type was B3 with 81%, followed by B1 and B2 (12%) and C (7%). The same was reported by Westerveld et al. [7].

Since 2011, the MIS technique is used as the preferred alternative to OS in patients with ankylosing spondylitis. The indication for OS or MIS depended on several factors, such as the severity of the injury or the surgeon's preference. More severe injuries tended to be treated more with OS technique. To detect a possible bias due to fracture severity based on the AOSpine classification, the individual fracture morphology of the patients was compared between both techniques.

Multi-level posterior fixation is considered the standard procedure for fractures of the thoracic and lumbar spine in patients with ASD [5, 11]. The aim of surgical fracture treatment in ASD has been described in the literature. Instrumentation (OS or MIS) and reduction should be performed to promote osseous healing and prevent further neurological deterioration [5, 11].

**Fig. 6:**
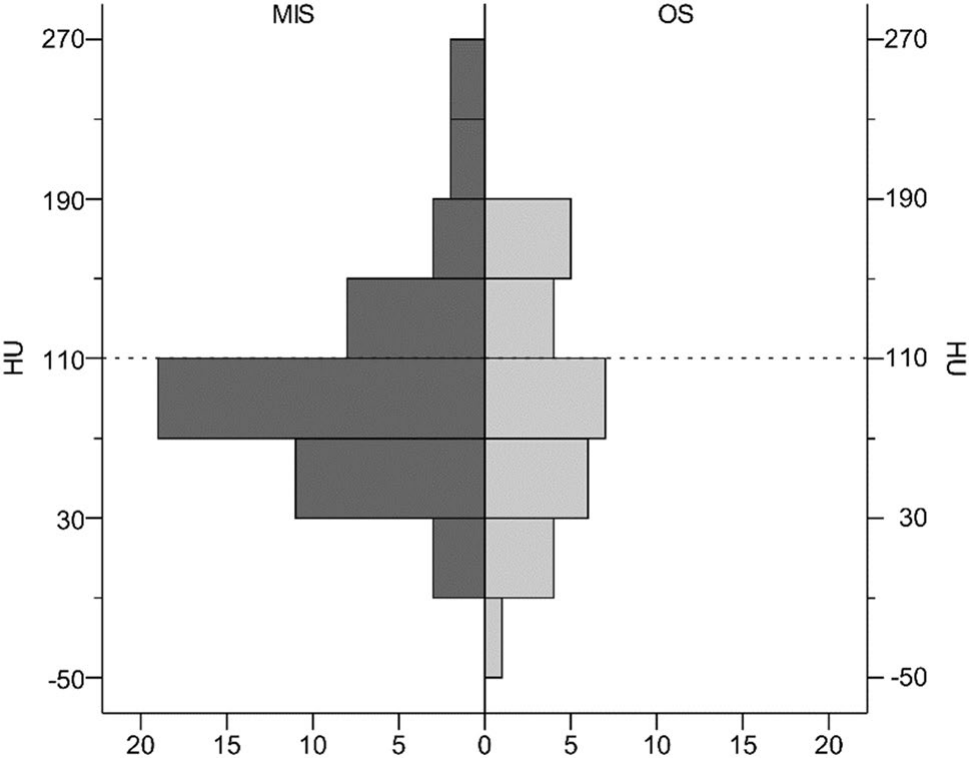
Distribution of Hounsfield Units (HU) for both groups (MIS and OS). The threshold for osteoporosis (HU < 110) is demonstrated as a dotted line

To our knowledge, there are no publications on the differences in CA in the MIS and OS groups of patients with ASD. We found no differences in the CA following reduction. Therefore, we cannot confirm our hypothesis that the OS technique offers advantages related to reduction. Overall, traumatic malalignment improved only marginally, regardless of the surgical approach used. A significant improvement in the primary malalignment was not observed in the MIS and OS groups. In both groups, the reduction effect of the fracture was low. Figure 6 shows a comparison of the achieved CAs. The different distributions based on the AOSpine classification in the MIS and OS groups had a significant influence on DApreop and DApostop. DApreop was not significantly different, but it tended to be more pronounced in the OS group. Surprisingly, in contrast with the hypothesis that the OS technique resulted in a better reduction, the DApostop was significantly greater in the OS group even after adjusting for the AOSpine classification. Thus, OS tended to be even worse than MIS, although CA showed no significant difference.

Despite all the reduction efforts, lordotic residual malalignment is often not preventable [5]. Lindtner et al. proposed a treatment concept involving percutaneous posterior instrumentation with less rigid rods and fracture reduction through postoperative mobilization based on the studies of 20 ASD patients with thoracolumbar fractures. The radiological follow-up after the mobilization showed that the pre-traumatic individual sagittal profile was restored spontaneously 3 weeks after MIS with soft rods and 6 months after OS with rigid rods [5]. We used only rigid rods. Taking these findings into account, the initially remaining lordotic malalignment due to the reduction failure seems not to be clinically relevant and can be considered to resolve after 6 months during follow-up.

Data on the prevalence of osteoporosis in ASD patients vary widely in the literature, ranging from 19 to 62% [3]. Assessments utilizing HU measurement allow reliable estimations of bone quality based on clinical CT data sets without the need for additional DXA or qCT [24]. Our data show a prevalence of 68% for HU below 110, and, thus, osteoporosis [3] which is consistent with the findings cited above.

Regarding poor bone quality, reduction maneuvers can lead to pull-out failure of pedicle screws and carry the risk of iatrogenic neurological deficits in these patients [5].

The screw position accuracy did not differ significantly in the OS and MIS groups (*p* = 0.095). The positioning of pedicle screws in patients with ASD is challenging because of the special anatomical conditions [25]. From our experience, it was expected that the visualization of anatomical landmarks would make the OS superior in terms of screw position. However, these findings cannot be confirmed. Although not significantly different (*p* = 0.095), the proportion of misplaced screws after OS (16%) was twice as high as that after MIS (8%).

In a 2015 study investigating 288 screws inserted on the thoracic spine during MIS and OS in 16 cadavers, Kwan et al. concluded that the positions of the pedicle screws during MIS and OS were comparable and without any significant differences [26]. Raley et al. retrospectively examined 424 MIS inserted thoracic and lumbar pedicle screws and reported a 10% frequency of malposition. However, only 13% of patients underwent surgery because of trauma [27]. Reports of malpositioning (Gertzbein and Robbins grade C, D, E) after OS ranges from 8 to 40% [28]. In this respect, the screw misplacement rate in our study was consistent with the reported findings. No neurological deterioration occurred due to screw malposition, but three revision surgeries were necessary because of anterior breakage. However, this should be considered and neglected. In a cadaver study, Vaccaro et al. described all the structures at risk in relation to anterior screw breakage [29].

OS usually lasts longer and is associated with greater blood loss and greater soft tissue alteration [7]. As expected, we were able to show that MIS causes less blood loss than OS. Grass et al. also reported a highly significant difference in perioperative blood loss (*p* < 0.005): 40 mL (10–90 mL) for MIS and 870 mL (570–1200 mL) for OS [30]. Grass et al. analyzed intraoperative blood loss via a suction device and postoperative blood loss via drains [30]. However, when documenting blood loss through the suction device, blood that is lost through the cover and surgical wipes is not taken into account. After MIS, we usually do not use drains, and postoperative blood loss cannot be recorded. A further difference between the study by Grass et al. and our study was that only short-segment fixations were included. The greater blood loss in our cohort seems to be understandable.

Kai et al. found a blood loss of 104 mL (range: unquantifiable to 480 mL) but did not describe the technique for calculating blood loss in detail [14]. In our study, we followed the calculation of blood loss by Gombotz et al. [21]. Through this, the perioperatively balanced hemoglobin value including the external blood loss (suction and wipes), and also the perioperative internal loss (hematomas in muscles and soft tissue) was recorded. Thus, we were able to analyze the total intravascular volume loss.

We also found fewer perioperative fluid requirements in MIS patients. Consequently, our results show the superiority of the MIS technique, especially given its perioperative process parameters.

The necessity of transfusion did not differ significantly in the groups. In percentages or amounts, significantly more RCC was transfused in the OS group. This finding is in line with the literature and reflects the greater soft tissue compromise during the OS approach [31].

Six patients had preoperative neurological symptoms. This represents 8% of all the patients examined, which is lower than that described in the literature. Westerveld et al. described preoperative neurological symptoms in 20/54 (37%) patients with ASD [7]. Our study excluded patients with cervical injuries. This may be the reason for the difference. Westerveld et al. did not specify the injuries that caused neurological symptoms [7]. Mortality during hospital stay was 0%, which was also lower than that reported in the literature. Westerveld et al. reported a 13% mortality rate [7].

Lindtner et al. reported a rate of 21.4% for wound healing complications in the OS group (14 patients), while the MIS group (6 patients) did not show any wound healing complications [5]. The MIS group in our study showed no surgical site infections. In 7% of our OS patients, surgical side infections were found. In these two cases, revision surgery was performed. This was even lower than that described by Lindtner et al.

Grass et al. showed the tendency of shorter CST for MIS (85 min [− 25 to 120 min]) compared with OS (100 min [− 45 to 240 min]) [30]. Our findings may support these findings (MIS 89 ± 44 min, OS 166 ± 96 min: *p* < 0.001). CST was not significantly influenced by the AOSpine distribution. Kai et al. reported posterior fixation through MIS in patients with ASD in 2018 [14]. They included nine patients in their study and a CST of 180 min for MIS (range: 92–340 min). The CST for MIS in our study was half as long as that reported by Kai et al. The authors did not report the number of screws implanted per surgery. The techniques evolve, which may be the rationale for this difference. A longer CST is associated with higher infection rates, wound healing disorders, and other complications [5].

The fluoroscopic duration between MIS and OS techniques did not differ (*p* = 0.691). This supports the thesis of a good fluoroscopic visualization of landmarks during MIS. Kai et al. reported a longer fluoroscopy duration during MIS [14]. We think that the altered anatomical surface of the posterior elements [25] in ASD patients, which impeded clinical orientation and fluoroscopic support, is also necessary for OS.

The two groups did not differ based on ICU stay. With an average of 5 days, our patients tended to stay for a shorter duration than, for example, that reported by Ull et al. (14 days) [32].

The duration of hospital stay was significantly influenced by the different distributions of the AOSpine classification. However, the stay also differed after adjustment for this difference and was 2–3 times significantly longer in the OS group. Kai et al. reported an average duration of hospital stay of 76 days in patients with MIS [14]. The patients stayed for 14 days in the MIS group, which was shorter.

The retrospective nature of this study may have led to selection bias. We could not report any follow-up findings of the patients, and it remains unclear what the impact on long-term follow-up is. No assessment scores could be obtained in the retrospective data analysis to assess the functional outcome. In this respect, the results should not be interpreted in terms of functional impact. Prospective data collection on functional outcome by scores and radiological follow-up would be very useful and would significantly improve the level of evidence in this field. To what extent our findings correlate with functional effects must be shown in further studies. Thus, further prospective data collection is recommended. Blood loss was not measured but calculated; therefore, these results have to be interpreted cautiously.

## Conclusion

OS and MIS show equally limited performance in terms of the extent of fracture reduction achieved and can be considered comparably safe with regard to the accuracy of implant positioning. The present study showed that the MIS technique was superior to OS in terms of perioperative parameters, considering the lower CST, shorter hospital stay and blood loss with comparable radiation exposure.

## Data Availability

The collected data in the form of tables are with the corresponding author and can be viewed if required. Not applicable.
